# Detection of Neutrophil Extracellular Traps by Flow Cytometry in Healthy Controls and Patients with Systemic Lupus Erythematosus

**DOI:** 10.3390/biom16101475

**Published:** 2026-10-10

**Authors:** Andrey P. Aleksankin, Anastasia S. Avdeeva, Yulia N. Gorbunova, Zhanna G. Verizhnikova, Liubov V. Kondratyeva, Alisa A. Tarasenko, Maria N. Babaeva, Natalya V. Nizyaeva

**Affiliations:** 1 FSBSI «Petrovsky National Research Centre of Surgery», 119435 Moscow, Russia; 2 V.A. Nasonova Research Institute of Rheumatology, 115522 Moscow, Russia

**Keywords:** flow cytometry, neutrophil extracellular traps, NETosis, systemic lupus erythematosus, citrullinated histone H3, systemic immune-inflammation index (SII)

## Abstract

Excessive induction of neutrophil extracellular traps (NETs) formation (NETosis) and clearance system dysfunction lead to autoantigen accumulation, initiating autoimmune cascades in immune-mediated inflammatory rheumatic diseases. This study aims to quantitatively determine NETs using multicolor flow cytometry to evaluate the pathogenic role of NETosis in systemic lupus erythematosus (SLE). The study enrolled 70 patients with a verified diagnosis of SLE (median age 38.0 [27.5; 46.0] years; 62 females, 8 males). The control group consisted of 20 sex- and age-matched healthy individuals. Laboratory evaluation included multicolor flow cytometry, enzyme-linked immunosorbent assay (ELISA), and indirect immunofluorescence assay. NETosis induction was performed using phorbol 12-myristate 13-acetate (PMA). The proportion of neutrophils undergoing NETosis following PMA stimulation was significantly higher in SLE patients than in healthy controls (69.3% versus 27.5%, respectively, *p* = 0.0006). Conversely, serum levels of citrullinated histone H3 (measured by ELISA) were significantly higher in healthy controls compared to SLE patients (*p* = 0.005). The systemic immune-inflammation index (SII) was substantially higher in the SLE group compared to the healthy control group (613.9 versus 27.5, *p* less than 0.05). Furthermore, the percentages of both spontaneous and PMA-induced NETs demonstrated significant differences in patients with severe disease courses. The developed methodological approach based on multiparametric flow cytometry serves as an objective monitoring tool and could be recommended for verifying inflammatory activity in patients with a long-standing history of SLE exceeding 2 years.

## 1. Introduction

The formation of extracellular net network-like conglomerates of native DNA loaded with an enzymatic pool by neutrophils is classified in modern literature as NETosis. The resulting filamentous matrices are referred to as neutrophil extracellular traps (NETs) [1]. Under physiological conditions, neutrophils constitute the body’s first line of defense against infection, implementing this defense through phagocytosis, degranulation, generation of ROS (reactive oxygen species), and secretion of cytokines and chemokines [2]. The phenomenon of NETosis is considered to be an uncontrolled activation of neutrophils with the formation of NETs, which occurs as a result of decondensation of nuclear chromatin and subsequent destruction of the cytoplasmic membrane [3]. Acting as the basic mechanism of neutrophil resistance to microbial invasion, NETs perform a critical function in the destruction of pathogenic microorganisms [4].

SLE is a convenient model for studying the role of NETosis in the development and course of autoimmune reactions [5,6]. Low-density granulocytes (LDGs) are detected in the circulating blood of patients with this pathology. This cell population is characterized by excessive synthesis of proinflammatory cytokines (in particular, type I interferon) and accelerated initiation of the NETosis cascade relative to normal mature neutrophils.

The biological rationale for NET production is primarily to ensure the elimination of infectious pathogens. However, to date, extensive data has been obtained indicating that the persistence of excessive NETs under the influence of physicochemical triggers, microorganisms, or endogenous factors can induce a hyperergic inflammatory response and exacerbate damage to target organs [7,8].

In immune-inflammatory rheumatic diseases (IVRD), NET overproduction acts as a factor in organ and tissue destruction, directly supporting local inflammation and potentiating thromboembolism. This cascade is initiated by the massive release of proteases and intracellular enzymes that activate the macrophage and lymphocyte components of the immune system, as well as the presentation of autoantigens that stimulate the synthesis of autoantibodies to decoy components [9,10]. Increased production of Myeloperoxidase and Proteinase 3 plays a base role in the pathogenesis of systemic vasculitis. Free nucleic acids and DNA molecules serve as the main autoantigens in systemic lupus erythematosus [11], and citrullinated histones can be considered novel neoepitopes inducing the synthesis of antibodies to citrullinated proteins (ACP) in rheumatoid arthritis [12].

Various methodological approaches to verifying NETs have been described in detail in the current literature [13,14,15,16]. Each has its own analytical advantages and limitations, as a result of which a single, regulated “gold standard” for determining the level of trap formation is currently lacking. Flow cytometry is an objective tool for the highly accurate quantitative detection of NETs, enabling multiparametric analysis of single cellular events. This approach is based on the application of cell-permeant DNA-binding fluorescent dyes, such as SYTO 13 and its structural analog LUCS 13. Cells positive for LUCS 13 and specific neutrophil granular enzymes are detected in the granulocyte fraction isolated from peripheral blood after the induction of trap formation with PMA. As demonstrated in the fundamental methodological work by Masuda et al., the number of such positive events quantified by flow cytometry accurately reflects the actual level of NETosis induction [3].

Data on the presence and biological significance of NETs in the peripheral blood of healthy individuals and patients suffering from IRRH remain controversial. They are largely determined by the clinical characteristics of the study cohort, as well as the laboratory methods used for neutrophil analysis [17].

A significant factor in the heterogeneity of SLE is the age-associated changes in the immune system described by Jarzebska N. et al. in 2025 and Montoya-Ortiz G. in 2013 [18,19]. On the one hand, granulocyte immune senescence alters the kinetics of NET formation and reduces DNA matrix clearance, prolonging the persistence of autoantigens [20]. On the other hand, age affects the intensity of interferon signaling, a key driver of NETosis in SLE [21,22]. These chronological features modulate the intensity of the inflammatory response and require differentiated analysis of NET formation biomarkers. These changes directly modify the severity of the autoimmune response, necessitating verification of the key role of NETosis in this pathology.

Therefore, the aim of this study is to quantitatively assess the ability of neutrophils to form NETs using multicolor flow cytometry to subsequently study its role in the pathogenesis of systemic lupus erythematosus.

## 2. Materials and Methods

### 2.1. Patients and Clinical Characteristics

The study included 70 patients with a verified diagnosis of systemic lupus erythematosus (SLE) established in accordance with the 2012 Systemic Lupus International Collaborating Clinics (SLICC) classification criteria [23]. The cohort presented a median age of 38.0 [27.5; 46.5] years and included 62 women and eight men. A formal sample size calculation was not performed due to the exploratory nature of the study. The sample size was determined by the availability of consecutive patients meeting the strict inclusion criteria. Patients were observed at the clinic of the V.A. Nasonova Research Institute of Rheumatology and signed informed consent to participate in the study. The study was approved by the local ethics committee (protocol no. 25 dated 12/23/2021, protocol no. 07 dated 03/13/2025). At the time of inclusion, the main clinical manifestations of SLE were skin lesions (74%), joint lesions (arthritis/arthralgia) (94%), lupus nephritis (44%), non-scarring alopecia (51%), and serositis (pleurisy/pericarditis) (40%). The overwhelming majority of patients (94%) had positive antinuclear factor (ANA), antibodies to double-stranded DNA (anti-dsDNA) (70%), and hypocomplementemia of C3 and/or C4 complement components (64%). Regarding therapy, 94% of patients received low-dose glucocorticoids (median dose 10.0 [6.25; 20.0] mg/day) in combination with hydroxychloroquine (HCQ) (72.8%) at a dose of 200 mg/day. SLE activity was assessed using the SLEDAI-2K index, where low activity was defined as a score ≤ 4 points, and moderate/high activity as a score > 4 points [23]. Organ damage was assessed using the SLICC/ACR damage index (SDI), with the threshold for damage verification set at ≥ 2 points [5,24]. The control group consisted of 20 age- and sex-matched healthy controls without history of rheumatic diseases, oncological pathology, or acute infections (Table 1).

### 2.2. Laboratory Assays and Flow Cytometry

Prior to study inclusion and during follow-up, patients underwent standard clinical, laboratory, and instrumental examinations recommended by the Russian Association of Rheumatologists [25]. For neutrophil isolation, 4 mL of peripheral blood collected in EDTA tubes was diluted 1:1 with phosphate-buffered saline (PBS) (Biolot, St. Petersburg, Russia), layered over a cold Ficoll gradient solution (PanEco, Moscow, Russia), and centrifuged at 3000 rpm for 30 min at room temperature. The mononuclear cell interphase ring was discarded, and the bottom pellet containing granulocytes and erythrocytes was collected. To obtain a pure granulocyte fraction, a rapid hypotonic erythrocyte lysis step was performed. The cell pellet was washed twice in PBS at 1700 rpm for 10 min, and the final cell suspension volume was adjusted to 1 mL using RPMI-1640 medium (PanEco, Russia).

To induce ex vivo NETosis, neutrophils were stimulated with 60 nM PMA (Macklin, Shanghai, China) in a CO_2_ incubator at 37 °C and 5% CO_2_ for 4 h. Following incubation, cells were washed with PBS (2000 rpm, 5 min) and resuspended in PBS containing 3% bovine serum albumin (BSA) (Proliant Biologicals, LLC, Ankeny, IA, USA). 

**Figure 1:**
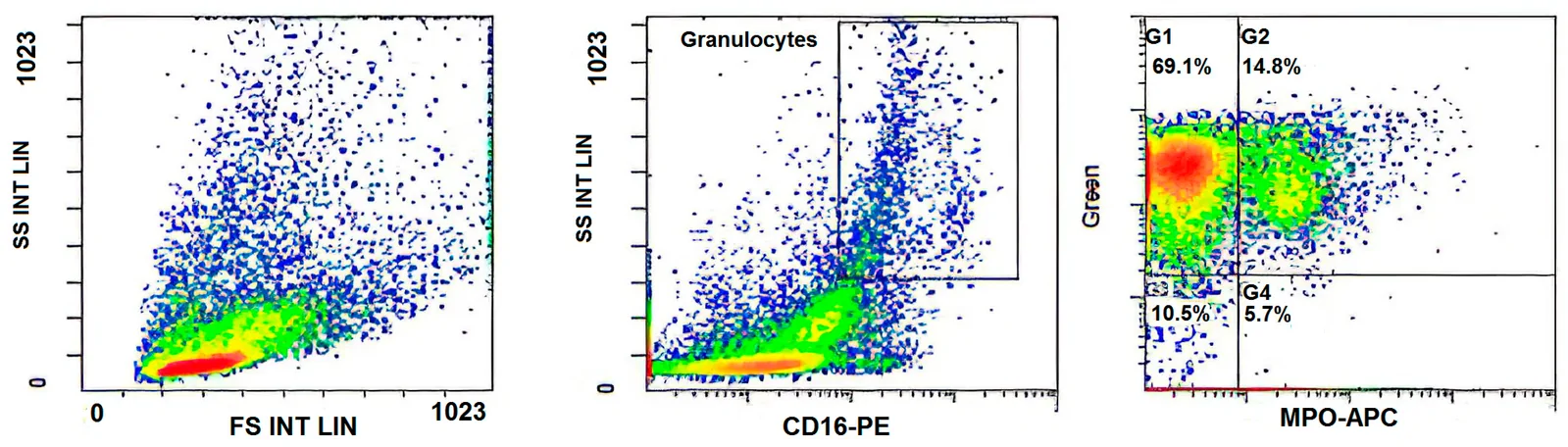
Multicolor flow cytometry protocol (for healthy controls).

**Figure 2:**
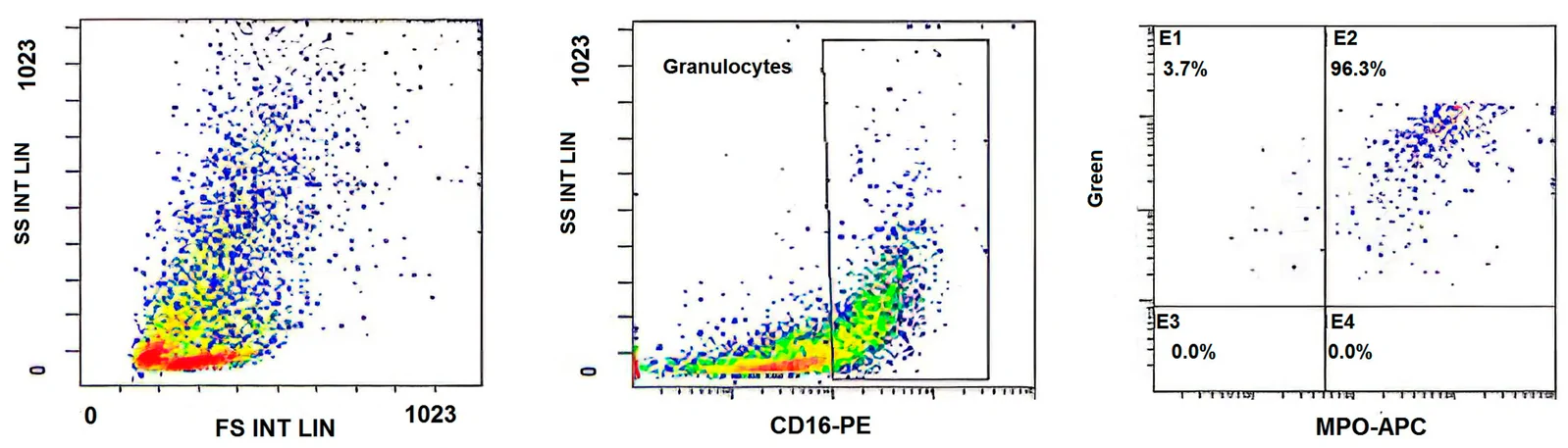
Multicolor flow cytometry protocol (for a patient with SLE).

For flow cytometry assessment, a 100 microliter cell suspension containing 10 to the power of 6 cells was incubated in the dark for 30 min with 10 microliters of anti-MPO-APC (Exbio, Praha-Vestec, Czech Republic) and 10 microliters of anti-CD16-PE (Beckman Coulter, Brea, CA, USA). After washing and centrifugation, the DNA-binding fluorescent nucleic acid dye LUCS 13 (Lumiprobe, Hunt Valley, MD, USA) was added at a final concentration of 1 μM. Cells were incubated for 10–20 min and analyzed using a Navios flow cytometer (Beckman Coulter, Brea, CA, USA). Granulocytes were gated based on forward and side scatter characteristics. Cells concurrently positive for anti-MPO-APC and LUCS 13 were identified as NETotic cells. The results were reported as the percentage (%) of all isolated neutrophils that were NETotic cells with and without PMA (Figure 1 and Figure 2).

The percentage of cells with signs of NETosis (positive for MPO and LUCS 13 Green) after PMA stimulation was 14.8%.

The percentage of cells with signs of NETosis (positive for MPO and LUCS 13 Green) after PMA stimulation was 96.3%.

The neutrophil-to-lymphocyte ratio (NLR) is the most popular marker of latent systemic inflammation and was calculated using the formula:

NLR = absolute neutrophils/absolute lymphocytes

The systemic immunoinflammatory index (SII) was calculated using the formula:

SII = absolute neutrophils × absolute platelets/absolute lymphocytes

### 2.3. Statistical Analysis

Statistical analysis was performed using the Statistica 10 software package (StatSoft Inc., Tulsa, OK, USA). Normality of data distribution was assessed using the Shapiro–Wilk test. Continuous variables were expressed as medians and interquartile ranges (IQR; 25th and 75th percentiles) due to non-normal distribution, and intergroup differences were analyzed using the Mann–Whitney U test. Categorical data are presented as frequencies and percentages. Spearman’s rank correlation coefficient (R) was used to assess relationships between clinical, demographic, and laboratory parameters, as well as NETosis markers. To control for multiple comparisons across correlation matrices, the Benjamini–Hochberg False Discovery Rate (FDR) procedure was applied, with statistical significance defined by q < 0.05. For individual intergroup tests, differences were considered statistically significant at *p* < 0.05.

## 3. Results

### 3.1. Evaluation and Selection of NETosis Inducers in SLE Patients

To select the most effective NETosis stimulator for the main study, the average percentage of NETosis cells in SLE patients was compared after exposure to PMA, lipopolysaccharide (LPS), and IgG. The analysis revealed that the average percentage of NETosis cells with PMA stimulation was 70%, while with LPS and IgG, these figures reached only 5% and 7%, respectively (Table 2).

Based on the obtained results, MA was selected for further work as the most effective NETosis stimulator.

### 3.2. Quantitative Analysis of NETosis Markers and Serum citH3 Levels in SLE Patients and Healthy Controls

The results of determining the percentage of NETosis in healthy controls and SLE patients are presented in Table 3. The percentage of cells with signs of NETosis after PMA stimulation in SLE patients (*n* = 70) was significantly higher than in healthy controls (*n* = 20) (*p* < 0.05) yielding 69.3% and 27.5%, respectively. CitH3, according to ELISA, was significantly higher in the group of healthy controls, –13.8% than in patients with SLE −9.85% (*p* < 0.05).

Although the number of cells with signs of NETosis without PMA stimulation and the anti-MPO level did not differ significantly between the groups (Table 2 and Table 3, Figure 1), according to IRIF, the p-ANCA titer was significantly higher in SLE patients (*n* = 18) compared to healthy controls (*n* = 3) (*p* < 0.05). The c-ANCA titer showed no significant differences between the groups. The NLR and SII results in patients with SLE were 2.6 (reference values < 3.0), within the normal range, and 613.9 (reference values ≤ 330), which is above the normal range.

When comparing the number of neutrophils with signs of NETosis after stimulation with PMA (%) in patients with SLE (*n* = 70) and healthy controls (*n* = 20), it was found that in SLE their number was 69.3 [39.1; 80.1], and in healthy controls 27.5 [12.0; 43.45].

### 3.3. Assessment of p-ANCA and c-ANCA Titers and Their Relationship with NETosis Processes

According to indirect immunofluorescence assay data (Table 4), patients with SLE showed a significant increase in p-ANCA titers to 20 [0; 80.0] versus 0 [0; 0] in healthy controls, while no significant intergroup differences were found in c-ANCA titers. The induction of p-ANCA in patients with SLE reflects a serological response to excessive extracellular release and prolonged tissue exposure of neutrophil granule antigens within extracellular traps, confirming the link between NETosis and systemic autoimmune inflammation.

### 3.4. Analysis of Integrated Hematological Indices of NLR and Systemic Immune-Inflammatory Index (SII) in SLE

Based on the evaluation of integrated hematological parameters (Table 5) in patients with SLE, statistically significant deviations from the reference values were identified. The median value of the systemic immunoinflammatory index in the overall cohort of patients was 613.9 [388.5; 916.6], which is multiple and statistically significantly higher than the upper limit of normal values (≤330). In contrast, the median neutrophil-lymphocyte ratio in the examined patients was within the reference range and was 2.6 [1.5; 3.7], which corresponds to the norm (<3). Thus, the overall cohort of patients with SLE was characterized by an isolated significant increase in the three-component SII index while maintaining the isolated NLR ratio.

### 3.5. Correlation Analysis of NETosis Parameters, Hematological Indices, and Clinical Laboratory Parameters in Patients with SLE

In the SLE cohort (Table 6), several robust correlations alongside exploratory trends were identified. Following FDR filtering, spontaneous NETosis showed a suggestive trend with CRP (r = 0.4136) and SDI (r = 0.3785). Conversely, induced NETosis maintained highly robust correlations with SLEDAI 2K (r = 0.3320), lupus nephritis (r = 0.3014), and ulcerative stomatitis (r = 0.2968) at the level of q < 0.05. Lupus nephritis also had an exploratory link with elevated NLR (r = 0.2745). For serological activity, anti-MPO levels demonstrated robust correlations with ANA (r = 0.3380), Ro (r = 0.3395), and La (r = 0.4021), while its association with anti-dsDNA (r = 0.2492) was exploratory. Inverse links of anti-MPO with C3 (r = −0.2551) and C4 (r = −0.2877) reflect complement depletion during immune complex fixation. Notably, the c-ANCA association with anti-dsDNA (r = 0.7748) was highly significant (q < 0.05). Finally, the inverse links of citH3 with SLEDAI 2K (r = −0.3171) and fraction C3 (r = 0.2994), alongside the negative SDI link with SII (r = −0.3051), remained statistically robust (q < 0.05), reflecting complex kinetics of citrullinated proteins during disease progression.

Considering the possible relationship between NETosis and SLE activity, all patients were divided into two groups based on SLEDAI 2K level. Patients with a SLEDAI 2K > 4 points were classified as having moderate/high SLE activity, and patients with SLEDAI 2K ≤ 4 points were classified as having low SLE activity. The results obtained between these two groups were then compared (Table 7).

### 3.6. Comparison of Clinical and Laboratory Parameters and NETosis Markers in Patients with SLE at Different Levels of Activity According to the SLEDAI 2K Scale

The percentage of neutrophils with signs of NETosis after PMA stimulation was significantly higher in patients with SLEDAI 2K > 4 (72.4%) than in patients with SLEDAI 2K ≤ 4 (60.2%), which allows us to consider this indicator as an additional marker for assessing SLE activity. The citH3 level was significantly lower in the group with moderate/high disease activity (9.4 ng/mL) compared to the low activity group (10.7 ng/mL). This fact is consistent with the previously described negative correlation of this marker with the SLEDAI 2K index. The NLR index values were statistically significantly higher in the cohort of SLE patients with SLEDAI 2K > 4 (2.96) than in the group with SLEDAI 2K ≤ 4 (2.3). This trend indicates a more pronounced systemic inflammatory response with increasing disease severity.

### 3.7. Comparative Analysis of Clinical and Laboratory Parameters of Neutrophil NETosis and Systemic Inflammation in Patients with SLE (n = 70) Depending on the SDI Damage Index 

In patients with severe organic lesions (SDI ≥ 2), a significant fourfold increase in the spontaneous NETosis rate was observed, reaching 9.4% [3.8; 18.9] compared to 2.3% [1.5; 4.4] in patients with minimal or no lesions (SDI < 2) (Table 8). The integrated hematological index (SII) in the subgroup of patients with SDI ≥ 2 demonstrated significantly lower values, amounting to 435 [351; 859] versus 745 [410; 1091] in the subgroup of individuals with IP SDI < 2.

Acute SLE implies the onset of the disease with pronounced clinical manifestations that are clearly specific for SLE, while a chronic/subacute course is accompanied by an onset with a protracted, unclear clinical picture.

It remained unclear with which type of SLE course the maximum rates of NETosis were associated. To test this, patients were divided into two groups based on their SLE course: with an acute course and with a chronic/subacute course. The results obtained between the two groups were then compared.

### 3.8. Comparative Analysis of Clinical and Laboratory Parameters and Markers of NETosis Depending on the Nature of the Course of SLE

A comparative analysis of NETosis parameters and hematological indices depending on the SLE course revealed three statistically significant differences (Table 9). In patients with an acute course of the disease, a significant increase in the level of induced NETosis was recorded to 73.4% [67.5; 82.7] versus 62.3% [36.7; 74.9] observed in patients with a chronic or subacute course of the process. The acute phase of the disease was characterized by a pronounced proinflammatory shift in integral blood indices. The median NLR in patients with an acute course was significantly higher, reaching 3.3 [2.8; 4.0] compared to 2.1 [1.5; 3.4] in the group with a chronic/subacute course. The systemic immunoinflammatory index in patients in the acute period also demonstrated an almost twofold increase, reaching a median of 866 [673; 998] versus 472 [362; 916] in patients with a chronic/subacute course of the disease.

Of the patients included in the study, 41 had a SLE duration of more than 2 years, and 29 had a disease duration of less than 2 years. Given the shorter duration of immunosuppressive therapy in the early SLE group, further analysis of this cohort is of great clinical interest (Table 10).

### 3.9. Correlation Analysis of NETosis Parameters and Clinical and Laboratory Parameters in Patients with SLE Duration Greater than 2 Years

In cases of patients with SLE duration > 2 years, spontaneous NETosis levels correlated positively with CRP (r = 0.3537) and SDI (r = 0.3752) (Table 10). Induced NETosis demonstrated direct associations with SLEDAI 2K (r = 0.3607), anti-dsDNA levels (r = 0.3877), lupus dermatitis (r = 0.3213), and lupus nephritis (r = 0.3121), as well as an inverse association with C3 levels (r = −0.3337). CitH3 levels correlated negatively with SLEDAI 2K (r = −0.3284) and showed a direct association with C3 (r = 0.3350). The anti-MPO level correlated positively with the anti-nuclear factor (ANA) titer (r = 0.4790) and antibodies to the Ro antigen (r = 0.5520). The c-ANCA titer demonstrated direct significant relationships with the anti-dsDNA level (r = 0.7488) and an inverse relationship with myositis (r = −0.7319), while a positive non-significant trend was observed towards the presence of nephritis (r = 0.5092, *p* = 0.161). The NLR index correlated positively with the anti-dsDNA level (r = 0.3374). All reported significant correlations successfully maintained statistical significance after Benjamini–Hochberg FDR correction at the q < 0.05 level.

### 3.10. Correlation Analysis of NETosis Parameters and Clinical and Laboratory Parameters in Patients with SLE Duration of No More than 2 Years

In patients with SLE duration of 2 years or less (Table 11), spontaneous NETosis was inversely correlated with the development of alopecia (r = −0.5322), while induced NETosis was directly correlated with aphthous stomatitis (r = 0.3879). A significant direct correlation was also found between spontaneous NETosis and complement C3 consumption (r = 0.4220). A strong direct correlation was found between the c-ANCA titer and the anti-dsDNA level (r = 0.7746), and between the p-ANCA titer and the presence of vasculitis/Raynaud’s (r = 0.7926). The NLR index was inversely correlated with anti-La (r = −0.4526), and the systemic SII index was inversely correlated with the accumulation of lesions according to the SDI (r = −0.3903).

### 3.11. Correlation Analysis Taking into Account Age, Duration of Medical History, and NETosis Indices in SLE

The age of patients suffering from SLE had a significant direct correlation with spontaneous NETosis (r = 0.3449), erythrocyte sedimentation rate (ESR, r = 0.3470), and CRP levels (r = 0.4136) (Table 12). A strong direct correlation was also found between age and disease duration, both at first symptom (r = 0.3840) and at official diagnosis (r = 0.3291). The anti-CL IgM serological profile showed a significant inverse correlation with patient age (r = −0.4259) and disease duration at official diagnosis (r = −0.3146).

## 4. Discussion

A methodological advantage of our study was the use of multicolor flow cytometry to detect NETs through the detection of the MPO-DNA complex [11]. According to study by Masuda S. et al. [3], flow cytometry is a highly objective and quantitative method, exceeding traditional image analysis in speed and reproducibility. In translational research settings, the transition to automated assessment of thousands of events helps minimize the subjectivity inherent in visual counting [26,27]. To confirm the functional responsiveness of this readout in our protocol, we evaluated the shift in the MPO+/LUCS13+ population upon direct PMA stimulation (Figure 1 and Figure 2). Nevertheless, we acknowledge that the use of cell-permeant dyes may carry inherent specificity limitations regarding the definitive differentiation of NETosis from other forms of cell death. To overcome this limitation and ensure even higher specificity, cell-impermeant nucleic acid stains, such as Sytox Green, will be incorporated into the flow cytometry panels in our future work.

The use of flow cytometry allowed us to record the significantly higher level of induced NETosis in patients with SLE −69.3%—compared to healthy controls –27.5% (*p* < 0.05). The increase in this indicator to 72.4% in patients with SLEDAI 2K > 4 confirms the prognostic value of NET assessment. This is consistent with the results obtained by Juo et al. and Lee et al. regarding the contribution of neutrophil traps to the development of systemic inflammation and vascular complications, including the risk of thrombosis [15,26].

The findings on the paradoxically low citH3 level in SLE patients (9.85 ng/mL) compared to the control group (13.8 ng/mL) (*p* < 0.05) are consistent with the results of Wang W. et al. [16] and can be explained by the specific organ damage in the study group. The presence of lupus nephritis in patients suggests active deposition of neutrophil trap components, including citH3, directly in renal tissue.

The obtained data on citH3 levels provide important confirmation of changes in the dynamics of NETosis during disease progression. Statistically, the median in citH3 level in the group of patients with SLEDAI 2K > 4 was 9.4 ng/mL versus 10.7 ng/mL in the group with SLEDAI 2K ≤ 4 (*p* = 0.005). This fact reflects the same pathogenic trend as the results of flow cytometry: with high activity of the process against the background of increased trap formation, accelerated elimination of modified histones from the bloodstream occurs.

A direct relationship between the molecular marker citH3 and the cellular response emphasizes the systemic nature of neutrophil activation with SLEDAI 2K > 4. This is consistent with the concept of the role of NETs as a source of citrullinated autoantigens [12] and the possibility of their rapid degradation or fixation in inflammatory foci during a pronounced immunopathological process [23,28]. According to the data of Gupta and Kaplan, excessive NET formation in SLE is often accompanied by impaired clearance, which contributes to the maintenance of inflammation in target tissues, including the kidneys [6].

The selection of the most effective NET formation stimulator for the main study was based on a comparative analysis of PMA, lipopolysaccharide, and IgG. PMA was found to induce NET formation in 70% of cells, while the efficacy of LPS (5%) and IgG (7%) was significantly lower. Potent activation of protein kinase C via PMA allows for a more complete assessment of the maximum functional potential of neutrophils to form NETs. This methodology is consistent with the data of El-Ghoneimy DH and Hesham M., emphasizing the key role of NADPH oxidase activity in the mechanisms of trap formation in SLE, and the work of Remijsen et al., indicating the association of NETosis with autophagy processes and superoxide generation [29,30].

Our finding of a significant increase in p-ANCA titers in patients with SLE compared to healthy controls (*p* = 0.046), along with the absence of significant changes in c-ANCA (*p* > 0.05), has a profound pathogenic basis. These results are consistent with the concept of systemic neutrophil priming, described in detail by Villanueva E. et al. [27]. The induction of p-ANCA, directed primarily against azurophilic granule antigens (in particular, myeloperoxidase), directly indicates that SLE patients mount a specific serological response to excessive extracellular chromatin release. Long-term tissue exposure of intracellular proteins transported on extracellular traps transforms them into highly immunogenic neoantigens recognizable by B lymphocytes.

As demonstrated in a study by Hakkim et al., a vicious cycle between uncontrolled NET formation and autoantibody induction is observed in SLE [28]. The resulting p-ANCAs are capable of binding to receptors on the membrane of activated neutrophils, triggering their repeated lysis via a positive feedback mechanism termed the “ANCA-NETosis vicious cycle” [31].

The significant (almost twofold) increase in the SII index observed in SLE patients compared to normal controls (*p* < 0.05) indicates high systemic inflammatory activity at the cellular level. This finding is fully consistent with the results of a meta-analysis by Lee Y. H. and Song G. G. [32], in which a comprehensive calculation of the neutrophil, platelet, and lymphocyte pool demonstrated higher sensitivity to latent endothelial damage and immune complex inflammation than standard isolated tests.

The NLR index in patients remained within the normal range despite a critical increase in SII, which has profound pathogenetic significance. The SII formula, in addition to leukocytes, takes into account the absolute platelet count. The recorded shift is due to the active involvement of the platelet component of hemostasis in the pathological process. As shown in studies by Lande R. et al. [33], activated platelets in SLE are capable of entering into direct intercellular interactions with primed neutrophils. This interaction induces the extracellular release of nuclear contents and triggers a cascade of immunothrombosis. Thus, the increase in the SII index in the examined patients reflects the association of thrombus formation, systemic autoimmune inflammation and reactive NETosis in the vascular bed.

Correlation analysis confirmed the essential contribution of NETosis to the development of systemic damage in SLE. The association of induced NETosis with the development of lupus nephritis (r = 0.3014) is consistent with data on the cytotoxic potential of extracellular traps, whose components induce podocyte apoptosis and damage the glomerular basement membrane [8]. Impaired mechanisms for the disposal and degradation of NET structures are directly linked to the severity of kidney damage and secondary autoimmune syndromes in the classic studies of Hakkim et al. [28] and Leffler J. et al. [9].

The strong positive correlation we found between c-ANCA titers and anti-dsDNA antibodies (r = 0.7748) supports the theory of coupled presentation of autoantigens within traps, as described in detail by Khandpur et al. [12], where DNA and granule enzymes are presented to immunocompetent cells simultaneously, triggering synergistic antibody synthesis. The negative correlation of anti-MPO levels with complement fractions C3 (r = −0.2551) and C4 (r = −0.2877) reflects the classical mechanism of complement system consumption during fixation of formed immune complexes against the background of high serological activity, which is confirmed by data from modern reviews on the pathogenesis of rheumatic diseases [1,6].

In turn, the direct relationship between spontaneous NETosis and CRP concentration (r = 0.4136) and damage accumulation according to the SDI index (r = 0.3752) [24] demonstrates that persistent neutrophil degranulation serves as a continuous trigger of sluggish endogenous inflammation [11], accelerating irreversible organ failure even outside of obvious clinical exacerbations.

An increase in induced NETosis during an exacerbation, verified by an increase in SLEDAI 2K scores, indicates underlying pathological activation and high reactivity of circulating granulocytes. Under the influence of excess systemic cytokines and immune complexes, cells enter a hypersensitive state, responding to stimuli with excessive release of traps [34].

The presence of concomitant systemic inflammation is confirmed by a synchronous increase in the NLR hematological index. A decrease in serum concentrations of the citH3 marker against the background of high pathological process activity according to the SLEDAI 2K score indicates the phenomenon of active “consumption” of this protein. Being a potent neoantigen, the released citrullinated histone is quickly bound by antibodies to circulating immune complexes and deposited directly in target organs [35]. Tissue fixation of macromolecules leads to a predictable decrease in the detectable free antigen fraction in the systemic circulation.

A significant and pronounced increase in the percentage of cells involved in spontaneous NETosis without exogenous stimulation in vitro in patients with SDI ≥ 2 serves as a key marker of the advanced stage of the disease. This fact indicates a state of deep initial priming of circulating neutrophils induced by persistent autoimmune inflammation. As noted in their studies by Villanueva E. et al. and Hakkim A. et al., in the chronic, torpid course of SLE, the continuous formation of NETs and the uncontrolled release of toxic granule enzymes into the vascular bed directly contribute to endothelial destruction and the progression of irreversible organic changes [27,28].

The paradoxical decrease in the SII index observed in the most severe patients with an SDI ≥ 2 has a pathogenetic and clinical explanation. The SII index reflects the balance of circulating neutrophils, platelets, and lymphocytes. In the late stages of the disease, associated with a high organ damage index, activated blood cells migrate intensively and are deposited directly in target tissues, where their subsequent spontaneous death occurs. Furthermore, this cohort of patients traditionally receives the most aggressive regimens of prolonged immunosuppressive therapy. According to data published in 2011 by Lande R. et al., massive cytostatic and glucocorticoid therapy has a pronounced suppressive effect on bone marrow hematopoiesis, which naturally reduces the pools of circulating cells in the systemic circulation and mathematically underestimates the integral indices, masking ongoing tissue damage [33].

A significant increase in induced NETosis in patients with acute SLE indicates a significantly higher functional reserve and cytolytic reactivity of the circulating neutrophil pool during the acute phase of the disease. This phenomenon is consistent with the concept of “cytokine priming” described by Knight J. S. et al., according to which high concentrations of systemic proinflammatory interleukins and interferon-alpha during the acute phase activate neutrophils into a hyperreactive state [35]. When exposed to a laboratory stimulus in vitro, this leads to a massive, explosive release of extracellular traps, which is accompanied by a synchronous increase in the NLR index. The synchronous and highly significant increase in the hematological indices NLR (*p* = 0.0076) and SII in patients with an acute course reflects the classic inflammatory leftward shift in the leukocyte count.

As demonstrated ina study by Soliman W. M. et al., a critical increase in the SII index during the acute phase of rheumatic diseases indicates a conjugate hyperactivation of not only the granulocyte but also the platelet component of hemostasis [36]. During the acute phase, platelets actively cooperate with primed neutrophils, enhancing their ability to generate traps directly in the vascular bed. Thus, the combined increase in the functional potential of NETosis and the NLR/SII index serves as a specific pathogenetic marker of the high intensity of the systemic immune-inflammatory response during the acute phase of SLE.

The transformation of the role of neutrophils is clearly evident when analyzing the duration of the disease. In the early period of SLE (≤ 2 years), NETosis mainly correlates with lupus nephritis (r = 0.3663), ulcerative stomatitis (r = 0.3879) and complement C3 consumption (r = 0.4220) [5,27,29], and the p-ANCA titer becomes an indicator of vascular disorders (r = 0.7926) [6]. With an SLE duration of > 2 years, NETosis parameters and the citH3 level are transformed into indicators of accumulated organ damage, correlating with the SDI index (r = 0.3752), and become closely associated with the level of antibodies to dsDNA (r = 0.3877) and activity on the SLEDAI 2K scale (r = 0.3607) [28].

The identified relationship between age, systemic inflammation markers, and neutrophil function reflects the combined effects of prolonged systemic lupus erythematosus and immune senescence.

The direct correlation between age and spontaneous NETosis indicates that neutrophils in older patients are initially in a state of profound biological priming. This is consistent with the fundamental concept of age-associated inflammation published by Franceschi C. et al. in 2018, according to which aging is accompanied by persistent subclinical activation of innate immune cells [22]. With increasing age, the membrane potential and metabolism of granulocytes undergo qualitative changes, leading to their accelerated, uncontrolled spontaneous destruction with the release of NETs, even in the absence of additional microbial or chemical stimuli [20,21]. In 2016, Gupta and Kaplan confirmed that PMA-induced NETosis has been confirmed to be not age-related [6].

This suggests that the preservation of the maximum functional reserve of cells in young patients, in whom NETosis is vigorously initiated upon stimulation, while in older patients this reserve is already partially depleted due to the constant spontaneous secretion of DNA matrix into the blood flow.

This study features several limitations. First, it utilizes a single-center design, which may limit the generalizability of the findings to wider SLE populations. Second, the sample size (*n* = 70 patients and *n* = 20 controls) remains relatively small for extensive subgroup calculations. Finally, the potential confounding effects of age, disease duration, and ongoing immunosuppressive treatments (glucocorticoids and hydroxychloroquine) on neutrophil activation loops cannot be excluded and require further investigation in larger cohorts.

## 5. Conclusions

Multicolor flow cytometry after PMA stimulation demonstrated high efficiency for quantifying neutrophil extracellular traps, demonstrating a significant difference between groups and linking NETosis with immunological and clinical abnormalities in SLE. High disease activity and the development of nephritis were found to be associated with the accelerated “consumption” of citrullinated histone H3, its fixation into immune complexes, and its deposition in renal tissue. The role of NETosis markers changes with increasing disease duration: in the early period (up to 2 years), they correlate with the manifestation of organ pathology, while in the long-term course, they become indicators of accumulated damage according to the SDI index. Thus, the study results demonstrated the significant contribution of NETosis to the development of SLE. The next step is to standardize NETosis assessment methods for the subsequent implementation of these markers in clinical practice and the development of targeted therapy based on the obtained data.

## Figures and Tables

**Table 1:** Demographic and clinical characteristics of SLE patients and healthy controls.

Parameter	SLE Patients (*n* = 70)	Healthy Controls (*n* = 20)	*p*-Value
Age, years, Median [Q25; Q75]	38.0 [27.5; 46.5]	36.5 [29.0; 43.0]	0.64
Sex, female/male, *n*	62/8	18/2	1.00
**Clinical Manifestations, *n* (%)**			
Skin lesions	52 (74%)	0 (0%)	<0.001
Joint lesions (arthritis/arthralgia)	66 (94%)	0 (0%)	<0.001
Lupus nephritis	31 (44%)	0 (0%)	<0.001
Non-scarring alopecia	36 (51%)	0 (0%)	<0.001
Serositis (pleurisy/pericarditis)	28 (40%)	0 (0%)	<0.001
**Laboratory Findings**			
Positive ANA, *n* (%)	66 (94%)	0 (0%)	<0.001
Anti-dsDNA positive, *n* (%)	49 (70%)	0 (0%)	<0.001
Hypocomplementemia (C3 and/or C4), *n* (%)	45 (64%)	0 (0%)	<0.001
**Disease Activity Indices**			
SLEDAI-2K, points, Median [Q25; Q75]	6.0 [4.0; 12.0] *	0.0 [0.0; 0.0]	<0.001
Low activity (≤4 points), *n* (%)	24 (34.3%)	20 (100%)	<0.001
Moderate/high activity (>4 points), *n* (%)	46 (65.7%)	0 (0%)	<0.001
SLICC/ACR Damage Index (SDI) >=2, *n* (%)	18 (25.7%)	0 (0%)	<0.001
**Concomitant Therapy, *n* (%)**			
Low-dose glucocorticoids	66 (94%)	0 (0%)	<0.001
Median dose, mg/day [Q25; Q75]	10.0 [6.25; 20.0]	0	-
Hydroxychloroquine (HCQ)	51 (72.8%)	0 (0%)	<0.001
Dose 200 mg/day	51 (72.8%)	0 (0%)	<0.001

Note: Data are expressed as Median [25th; 75th percentiles] representing the interquartile range (IQR). Differences with *p* < 0.05 were considered statistically significant. Intergroup differences were analyzed using the Mann–Whitney U test. ANA—antinuclear factor; anti-dsDNA—antibodies to double-stranded DNA; SLEDAI-2K—Systemic Lupus Erythematosus Disease Activity Index 2000; SLICC/ACR—Systemic Lupus International Collaborating Clinics/American College of Rheumatology damage index. * — calculated exclusively for the SLE patient cohort.

**Table 2:** Comparative analysis of the efficacy of various activators of extracellular DNA release (LPS, IgG, and PMA) in SLE patients.

Evaluated Parameter	Lipopolysaccharide (LPS)	Immunoglobulin G (IgG)	Phorbol 12-Myristate 13-Acetate (PMA)
Mean percentage of NETotic cells, %	5	7	70

**Table 3:** Comprehensive clinical and laboratory assessment of NETosis parameters and ANCA profile in patients with SLE compared to healthy controls.

Parameter/Marker	Healthy Control (*n* = 20)	Patients with SLE (*n* = 70)	*p*-Value
Spontaneous neutrophil NETosis level, %	2.5 [1.7; 2.8]	2.9 [1.6; 7.6]	>0.05
Level of induced (PMA) NETosis, %	27.5 [12.0; 43.4]	69.3 [39.1; 80.1]	0.0006
Serum citH3 concentration, ng/mL	13.80 [12.40; 14.60]	9.85 [8.20; 11.40]	0.005
c-ANCA level (cytoplasmic type), titer	20.0 [0.0; 20.0]	20.0 [20.0; 40.0]	>0.05
p-ANCA level (perinuclear type), titer	0.0 [0.0; 0.0]	20.0 [0.0; 80.0]	<0.05

Note: Data are expressed as Median [25th; 75th percentiles] representing the interquartile range (IQR). Differences with *p* < 0.05 were considered statistically significant. Intergroup differences were analyzed using the Mann–Whitney U test, and statistical significance of correlations was calculated using Spearman’s rank correlation test.

**Table 4:** Comparative analysis of indirect immunofluorescence assay results in patients with SLE and healthy controls.

Evaluated Marker	Main Group (Patients with SLE)	Control Group (Healthy Controls)
	Value, Median [25th; 75th Percentile]	
c-ANCA, titer	20 [20; 40]	20 [0; 20]
pANCA, titer	20 [0; 80] *	0 [0; 0]

Note: Data are expressed as Median [25th; 75th percentiles] representing the interquartile range (IQR). Differences with *p* < 0.05 were considered statistically significant. Intergroup differences were analyzed using the Mann–Whitney U test, and statistical significance of correlations was calculated using Spearman’s rank correlation test. * — *p* < 0.05 compared to healthy controls.

**Table 5:** Results of assessment of the hematological index NLR and systemic inflammatory index SII in patients with SLE.

Indicator	Patients with SLE, Value, Median [25th; 75th Percentiles]
SII	613,9 [388,5; 916,6] *
NLR	2,6 [1,5; 3,7]

Note: Data are expressed as Median [25th; 75th percentiles] representing the interquartile range (IQR). Differences with *p* < 0.05 were considered statistically significant. Intergroup differences were analyzed using the Mann–Whitney U test, and statistical significance of correlations was calculated using Spearman’s rank correlation test. * — *p* < 0.05 compared to reference values.

**Table 6:** Spearman’s rank correlation analysis of NETosis parameters, hematological indices, and clinical laboratory markers in patients with SLE (*n* = 70).

Indicator	Spontaneous NETosis, %	Induced NETosis, %	citH3, ng/mL	anti-MPO, U/mL	c-ANCA, titer	p-ANCA, titer	NLR	SII
anti -dsDNA	0.0725	0.2082	−0.2009	0.2492 *	0.7748 *	−0.1385	0.1739	0.1203
anti -La	−0.0063	0.2421	0.1108	0.4021 *	−0.1709	−0.5655	−0.2357	−0.2367
CRP	0.4136 *	0.0174	−0.2118	0.1744	0.2958	0.2387	0.2256	0.1937
SDI	0.3785 *	−0.0475	0.0760	0.0131	−0.3683	0.2523	−0.1282	−0.3051 *
anti-Ro	0.0238	0.1238	−0.0254	0.3395 *	−0.1176	−0.4536	−0.2314	−0.1937
ANA	−0.1181	−0.2559	0.0677	0.3380 *	0.1312	−0.1524	0.0071	0.1502
SLEDAI 2K	−0.1189	0.3320 *	−0.3171 *	0.0499	0.2775	−0.0501	0.2008	0.0961
C3	0.1524	−0.2139	0.2994 *	−0.2551 *	−0.4641	0.0076	−0.0105	0.0835
C4	0.1106	−0.1327	0.0965	−0.2877 *	−0.1477	−0.2904	−0.0121	0.1397
Lupus nephritis	−0.0950	0.3014 *	−0.0446	−0.0193	0.1822	−0.2903	0.2745 *	0.1903
Ulcerative stomatitis	0.0501	0.2968 *	0.0164	−0.2310	−0.0272	−0.3432	−0.1819	−0.0181

Note: Data are expressed as Median [25th; 75th percentiles] representing the interquartile range (IQR). Differences with *p* < 0.05 were considered statistically significant. Intergroup differences were analyzed using the Mann–Whitney U test, and statistical significance of correlations was calculated using Spearman’s rank correlation test. * — *p* < 0.05 (statistically significant correlation).

**Table 7:** Comparison of clinical and laboratory parameters in patients with SLE at different levels of activity according to the SLEDAI 2K scale.

Evaluation Parameters	Patients with Active Course (SLEDAI 2K > 4)	Patients with Low Activity/Remission (SLEDAI 2K ≤ 4)
	Value, Median [25th; 75th Percentile]	
Baseline NETosis without stimulation (%)	2.3 [1.5; 7.8]	3.8 [2.1; 7.6]
NETosis level after exposure to PMA (%)	72.4 [51; 83.2] *	60.2 [37.4; 72.8]
Serum citH3 protein level (ng/mL)	9.4 [8.2; 10.6] *	10.7 [8.9; 12.9]
Integral Inflammation Index SII	751 [435; 1091]	483 [362; 898]
Index NLR	2.96 [2; 4.2] *	2.3 [1.47; 3.16]
Anti-MPO concentration (U/mL)	0.01 [0.003; 0.07]	0.007 [0.004; 0.05]

Note: Data are expressed as Median [25th; 75th percentiles] representing the interquartile range (IQR). Differences with *p* < 0.05 were considered statistically significant. Intergroup differences were analyzed using the Mann–Whitney U test, and statistical significance of correlations was calculated using Spearman’s rank correlation test. * — *p* < 0.05 compared to the low activity/remission group.

**Table 8:** Comparative analysis of clinical and laboratory parameters of neutrophil NETosis and systemic inflammation in patients with SLE (*n* = 70) depending on the SDI damage index.

Indicators and Markers (N = 70)	Patients with SDI ≥ 2 (*n* = 19),	Patients with SDI < 2 (*n* = 51)
	Value, Median [25th; 75th Percentile]	
Spontaneous NETosis, %	9.4 [3.8; 18.9] *	2.3 [1.5; 4.4]
Induced NETosis, %	68.9 [32.5; 72.7]	69.9 [38.3; 81.3]
citH3, ng/mL	9.8 [7.3; 11.6]	9.9 [8.4; 11.4]
Anti-MPO, U/mL	0.016 [0.002; 0.100]	0.007 [0.003; 0.057]
NLR	1.9 [1.5; 3.1]	2.8 [1.5; 4.1]
SII	435 [351; 859] *	745 [410; 1091]

Note: Data are expressed as Median [25th; 75th percentiles] representing the interquartile range (IQR). Differences with *p* < 0.05 were considered statistically significant. Intergroup differences were analyzed using the Mann–Whitney U test, and statistical significance of correlations was calculated using Spearman’s rank correlation test. * — *p* < 0.05 compared to patients with SDI < 2.

**Table 9:** Comparative analysis of clinical and laboratory parameters of neutrophil NETosis and systemic inflammation in patients with SLE (*n* = 70) depending on the course of the disease.

Neutrophil Activity and Inflammation Indices (N = 70)	Patients with Acute Form of the Disease (*n* = 16)	Patients with Chronic/Subacute Course (*n* = 54)
	Value, Median [25th; 75th Percentile]	
Spontaneous neutrophil NETosis, %	2.0 [1.5; 7.8]	3.3 [1.9; 7.1]
Induced neutrophil NETosis, %	73.4 [67.5; 82.7] *	62.3 [36.7; 74.9]
Serum citH3 level, ng/mL	9.8 [8.2; 10.8]	9.9 [8.2; 11.6]
Anti-MPO titer, U/mL	0.005 [0.003; 0.020]	0.010 [0.003; 0.070]
Hematological index NLR	3.3 [2.8; 4.0] *	2.1 [1.5; 3.4]
Integral index SII	866 [673; 998] *	472 [362; 916]

Note: Data are expressed as Median [25th; 75th percentiles] representing the interquartile range (IQR). Differences with *p* < 0.05 were considered statistically significant. Intergroup differences were analyzed using the Mann–Whitney U test, and statistical significance of correlations was calculated using Spearman’s rank correlation test. * — *p* < 0.05 compared to the chronic/subacute course group.

**Table 10:** Correlation analysis for SLE duration > 2 years (*n* = 41, * for ANCA *n* = 9).

Indicator	Spontaneous NETosis, %	Induced NETosis, %	citH3, ng/mL	anti-MPO, U/mL	c-ANCA, Titer	Index NLR
anti-dsDNA	0.0985	0.3877 *	−0.1494	0.2646	0.7488 *	0.3374 *
Myositis	−0.1661	−0.1902	0.1395	−0.0117	−0.7319 *	0.5472
anti-Ro	0.2382	0.0487	−0.1915	0.5520 *	0.0772	−0.1701
ANA	−0.1207	−0.0639	0.0898	0.4790 *	0.0786	0.0210
Lupus dermatitis	0.0888	0.3213 *	−0.2602	0.1128	0.3904	−0.5698
Activity SLEDAI 2K	−0.0262	0.3607 *	−0.3284 *	0.1352	−0.0093	0.3096
CRP	0.3537 *	0.0516	−0.1279	0.1962	0.1514	0.2943
Damage index SDI	0.3752 *	0.0003	0.1539	−0.0761	0.0914	−0.1686
Component C3	0.0985	−0.3337 *	0.3350 *	−0.2360	0.2478	−0.2189
Lupus nephritis	−0.1975	0.3121 *	−0.2109	−0.0114	0.5092	0.1362

Note: Data are expressed as Median [25th; 75th percentiles] representing the interquartile range (IQR). Differences with *p* < 0.05 were considered statistically significant. Intergroup differences were analyzed using the Mann–Whitney U test, and statistical significance of correlations was calculated using Spearman’s rank correlation test. * — *p* < 0.05 (statistically significant correlation).

**Table 11:** Correlation analysis for SLE duration ≤ 2 years (*n* = 29, * for ANCA *n* = 9).

Indicator	Spontaneous NETosis, %	Induced NETosis, %	anti-MPO, U/mL	c-ANCA, Titer	p-ANCA, Titer	NLR	SII
SDI	0.1259	−0.0907	−0.0816	−0.5952	0.5799	−0.2668	−0.3903 *
anti-dsDNA	−0.1373	−0.1307	0.2116	0.7746 *	0.0866	−0.0937	−0.0784
Lupus nephritis	−0.0524	0.3663	−0.1587	−0.1952	−0.7601 *	0.1787	0.0804
Vasculitis/ Raynaud’s	0.3814	−0.0715	0.2114	−0.1890	0.7926 *	−0.0179	−0.1429
Alopecia	−0.5322 *	−0.0092	0.2176	0.0000	0.0000	0.0461	0.0277
anti-La	−0.3107	0.1227	0.3749	−0.2155	−0.3935	−0.4526 *	−0.1992
Aphthous stomatitis	−0.1612	0.3879 *	−0.3553	0.0000	0.0000	−0.1401	−0.0216
C3	0.4220 *	−0.1640	−0.1669	−0.6362	−0.0606	0.2369	0.2379

Note: Data are expressed as Median [25th; 75th percentiles] representing the interquartile range (IQR). Differences with *p* < 0.05 were considered statistically significant. Intergroup differences were analyzed using the Mann–Whitney U test, and statistical significance of correlations was calculated using Spearman’s rank correlation test. * — *p* < 0.05 (statistically significant correlation).

**Table 12:** Correlation analysis taking into account age, duration of medical history, and NETosis indices in SLE (*n* = 70).

Indicator	Age	Duration of the First Symptom	Assessment of the Duration of the Disease	Spontaneous NETosis, %	Induced NETosis, %
Age		0.3840 *	0.3291 *	0.3449 *	−0.0914
Duration of 1 symptom	0.3840 *	1.0000	0.8783 *	0.2571	0.1328
Assessment of the duration of the disease	0.3291 *	0.8783 *	1.0000	0.2292 *	0.2158
Erythrocyte sedimentation rate	0.3470 *	0.0591	0.0183	−0.0914	0.0807
CRP	0.4136 *	0.1721	0.1759	0.1102 *	0.0233
anti-CL IgM	−0.4259 *	−0.2801	−0.3146 *	0.2175	0.0678

Note: Data are expressed as Median [25th; 75th percentiles] representing the interquartile range (IQR). Differences with *p* < 0.05 were considered statistically significant. Intergroup differences were analyzed using the Mann–Whitney U test, and statistical significance of correlations was calculated using Spearman’s rank correlation test. * — *p* < 0.05 (statistically significant correlation).

## Data Availability

The data presented in this study are available upon request from the corresponding author.

## Abbreviations

The following abbreviations are used in this manuscript:

ANCA	Antineutrophil cytoplasmic antibodies
c-ANCA	Cytoplasmic antineutrophil cytoplasmic antibodies
p-ANCA	Perinuclear antineutrophil cytoplasmic antibodies
ACP	Antibodies to citrullinated proteins
ANA	Anti-nucleated factor
BSA	Bovine serum albumin
citH3	Citrullinated histone H3
CRP	C-reactive protein
dsDNA	Double-stranded DNA
ELISA	Enzyme-linked immunosorbent assay
ESR	Erythrocyte sedimentation rate
IVRD	Immune-inflammatory rheumatic diseases MNO
HCQ	Hydroxychloroquine
MNO	Myeloperoxidase
LDGs	Low-density granulocytes
NETs	Neutrophil extracellular traps
NLR	Neutrophil lymphocyte ratio
PBS	Phosphate-buffered saline
PR3	Proteinase 3
PBMCs	Peripheral blood mononuclear cells
PMA	Phorbol 12-myristate 13-acetate
SDI	SLICC/ACR damage index
SLE	Systemic lupus erythematosus
SII	Systemic immune-inflammation index
